# Precision metagenomics sequencing for food safety: hybrid assembly of Shiga toxin-producing *Escherichia coli* in enriched agricultural water

**DOI:** 10.3389/fmicb.2023.1221668

**Published:** 2023-08-31

**Authors:** Meghan Maguire, Padmini Ramachandran, Sandra Tallent, Mark K. Mammel, Eric W. Brown, Marc W. Allard, Steven M. Musser, Narjol González-Escalona

**Affiliations:** ^1^Center for Food Safety and Applied Nutrition, Office of Regulatory Science, College Park, MD, United States; ^2^Office of Applied Research and Safety Assessment, Food and Drug Administration, College Park, MD, United States

**Keywords:** foodborne pathogens, *Escherichia coli*, nanopore sequencing, short-read sequencing, pre-harvest agricultural water, metagenomics, hybrid assembly

## Abstract

Culture-independent metagenomic sequencing of enriched agricultural water could expedite the detection and virulotyping of Shiga toxin-producing *Escherichia coli* (STEC). We previously determined the limits of a complete, closed metagenome-assembled genome (MAG) assembly and of a complete, fragmented MAG assembly for O157:H7 in enriched agricultural water using long reads (Oxford Nanopore Technologies, Oxford), which were 10^7^ and 10^5^ CFU/ml, respectively. However, the nanopore assemblies did not have enough accuracy to be used in Single Nucleotide Polymorphism (SNP) phylogenies and cannot be used for the precise identification of an outbreak STEC strain. The present study aimed to determine the limits of detection and assembly for STECs in enriched agricultural water by Illumina MiSeq sequencing technology alone, followed by establishing the limit of hybrid assembly with nanopore long-read sequencing using three different hybrid assemblers (SPAdes, Unicycler, and OPERA-MS). We also aimed to generate a genome with enough accuracy to be used in a SNP phylogeny. The classification of MiSeq and nanopore sequencing identified the same highly abundant species. Using the totality of the MiSeq output and a precision metagenomics approach in which the *E. coli* reads are binned before assembly, the limit of detection and assembly of STECs by MiSeq were determined to be 10^5^ and 10^7^ CFU/ml, respectively. While a complete, closed MAG could not be generated at any concentration, a complete, fragmented MAG was produced using the SPAdes assembler with an STEC concentration of at least 10^7^ CFU/ml. At this concentration, hybrid assembled contigs aligned to the nanopore-assembled genome could be accurately placed in a neighbor-joining tree. The MiSeq limit of detection and assembly was less sensitive than nanopore sequencing, which was likely due to factors including the small starting material (50 vs. 1 μg) and the dilution of the library loaded on the cartridge. This pilot study demonstrates that MiSeq sequencing requires higher coverage in precision metagenomic samples; however, with sufficient concentration, STECs can be characterized and phylogeny can be accurately determined.

## Introduction

Precision metagenomics is an approach that customizes the analysis of a metagenomic sample for the detection and classification of a specific pathogen. The development of culture-independent methods for the detection of foodborne pathogens can expedite source tracking and reduce prospective corrective measures during outbreak scenarios (Loman et al., 2013; Huang et al., 2017; Brown et al., 2019). For U.S. Food and Drug Administration (FDA)-designated zero-tolerance pathogens, such as *Listeria monocytogenes* and *Salmonella* spp., qPCR or metagenomic detection is sufficient to initiate microbiological isolate confirmation followed by regulatory action (Archer, 2018; CFSAN^1^). However, the pervasive nature of *Escherichia coli* necessitates further classification. There are more than 400 serotypes of Shiga toxin-producing *E. coli* (STECs) that can range in potential pathogenicity, as determined by the presence of a combination of *stx* (shiga toxin), *eae* (intimin), and other putative virulence genes (Kaper et al., 2004; Garmendia et al., 2005; Gonzalez-Escalona and Kase, 2019; Gonzalez-Escalona et al., 2019a,b; National Advisory Committee On Microbiological Criteria For Foods, 2019). STECs are responsible for ~2,400 hospitalizations per year causing severe diseases, ranging from diarrhea to hemolytic uremic syndrome (HUS) (Tarr et al., 2005; Mellmann et al., 2008; Scallan et al., 2011; Beutin and Martin, 2012).

Produce-related outbreaks have reportedly increased from 6% of all foodborne transmission in the 1990s to ~18% in a survey from 2003 to 2014 (Sivapalasingam et al., 2004; Fischer et al., 2015). Agricultural water has been considered a potential contamination source due to adjacent land use, incomplete water sanitization, or wild animal activity (FDA;^2^ Steele and Odumeru, 2004; Monaghan and Hutchison, 2012; Oliveira et al., 2012; Allende and Monaghan, 2015; Uyttendaele et al., 2015). Current STEC detection protocols as outlined by the FDA Bacteriological Analytical Manual (BAM) Chapter 4A (Feng et al., 2020) entail 24-h enrichment, qPCR detection of the *stx1, stx2*, and *wzy* genes of the O157 antigen, followed by several rounds of microbiological assays (TSAYE, TC-SMAC, and chromogenic agar plates), and single colony isolation used for whole-genome sequencing (WGS), which confirms the identity and pathogenic potential of the isolated STEC. This entire process can take ~2 weeks of analysis time, which may exceed the shelf life of produce and, in particular, leafy greens.

To expedite the analysis time, we have investigated culture-independent sequencing methods for the detection and classification of STECs directly in agricultural water. Long-read nanopore sequencing classified the highly abundant microbial community (>1% read abundance) but failed to accurately detect reads belonging to O157 in unenriched agricultural water due to low concentrations (Maguire et al., 2022). We have, therefore, suggested precision metagenomic analysis of enriched agricultural water instead, as the STEC concentration is increased to detectable levels by qPCR, according to the BAM Chapter 4A protocol. To investigate the use of long-read nanopore sequencing for precision metagenomics, it was necessary to establish the limits of detection and assembly of the technique (Maguire et al., 2021a). However, detection is not sufficient for the classification of the STEC serotype or virulotype. The limit of nanopore complete, fragmented assembly of a metagenome-assembled genome (MAG) was equivalent to the limit of qPCR detection at ~10^5^ CFU/ml. At STEC concentrations above ~10^7^ CFU/ml, a complete, closed O157:H7 MAG was obtained with the chromosome in single contig and the plasmid in the second contig (Maguire et al., 2021a).

Despite the benefits of nanopore sequencing, including affordability, portability, long reads, and real-time basecalling, the inherent error rate of nanopore sequencing (when using the fast-calling model) precludes the closed genomes from being used for phylogenic analysis. Short-read Illumina MiSeq sequencing technology, however, is highly accurate but creates a challenge in assembling highly repetitive regions, which can span several hundred base pairs (Bertrand et al., 2019; Gonzalez-Escalona et al., 2019a; Moss et al., 2020). Hybrid assembly using nanopore long-read sequencing as scaffolds and MiSeq for accuracy improves the overall outcome and generates closed or fragmented MAGs with enough quality to be used in phylogeny analysis (Gonzalez-Escalona and Sharma, 2020; Maguire et al., 2022). However, as determined for long-read sequencing, the limits of detection and assembly for the Illumina MiSeq technology have yet to be determined as well. We expected the limits of detection and assembly of Illumina MiSeq to be different from nanopore for several reasons: (1) the starting material is small (1–100 ng, depending on the DNA library preparation kit used vs. 1 μg for nanopore sequencing); (2) only a fraction of the library is used for sequencing, resulting in sampling bias (the entire material is used for nanopore sequencing instead); and (3) most MiSeq sequencing runs multiplex several samples (the exact level of detection or sequencing depth necessary for each sample has not been established). Therefore, we aimed to determine the limits of short-read Illumina MiSeq detection and MAG assembly using agricultural water artificially contaminated with STEC strain EDL933_2 (O157:H7), as previously determined for nanopore sequencing (Maguire et al., 2021a). We further aimed to test the use of hybrid assemblies for the recovery of a complete or fragmented O157:H7 MAG. For this last part, we have evaluated the performance of three hybrid assembly software (Unicycler, OPERA-MS, and SPAdes).

## Materials and methods

### Bacterial strains and media

We used a Shiga toxin-producing *E. coli* (STEC) EDL933 O157:H7 strain that was taken from our collection at CFSAN and is a variant of ATCC 43895 that has lost the *stx2* phage after several passages in the laboratory. We have termed this variant strain EDL933_2. EDL933_2 was grown overnight in static culture in tryptic soy broth (TSB) at 37°C.

### Short-read metagenomic sequencing, contigs, assembly, and annotation

For the short-read metagenomic studies using the MiSeq instrument, we used the same DNA extracted during our previous publication (Maguire et al., 2021a) and was used for determining both the detection and assembly limit for STEC EDL933_2 spiked into enrichments. The spiking experiment from that previous study was prepared as follows: 200 ml of an STEC-negative pre-harvest agricultural water sample was enriched according to the FDA BAM Chapter 4A protocol. An equal volume of 2× modified buffered peptone water with pyruvate (mBPWp) was added and incubated in static culture for 5 h at 37°C; then, an antimicrobial cocktail of acriflavin, cefsulodin, and vancomycin was added before an overnight static incubation (18–24 h) at 42°C. An equal volume (1 ml) of overnight-enriched agricultural water was artificially contaminated with 10-fold serial dilutions of an overnight culture (10^9^-10^5^ CFU/ml) of EDL933_2 diluted in TSB, resulting in a total of five samples (Water + Ecoli1–5). Additionally, a sample consisting only of the enriched agricultural water (Water) was used as a negative control for the presence of EDL933_2. The number of CFUs in the stock EDL933_2 spiked culture was calculated by spreading dilution on tryptic soy agar (TSA) plates. The DNAs were extracted from 1 ml of each artificially contaminated sample, Water and Water + Ecoli1–5, using the Maxwell RSC Cultured Cells DNA kit with a Maxwell RSC Instrument (Promega Corporation, Madison, WI) according to the manufacturer's instructions for Gram-negative bacteria with additional RNase treatment. DNA concentration was determined by a Qubit 4 Fluorometer (Invitrogen, Carlsbad, CA) according to the manufacturer's instructions. DNA quality was determined by the NanoDrop spectrophotometer (NanoDrop ND-1000 UV-Vis, Thermo Fisher Scientific, Waltham, MA) according to the manufacturer's instructions.

Short-read paired-end sequences were generated using Illumina MiSeq sequencing with a MiSeq V3 kit using 2 × 250 bp paired-end chemistry (Illumina Inc., San Diego, CA). DNA libraries were prepared using the DNA Prep kit with three samples in duplicate per cartridge according to the manufacturer's instructions. The starting material was 100 ng per sample, and 12 pmol was the final concentration loaded into the MiSeq instrument. A custom script (Supplementary Note) was developed to classify the reads by taxonomy using Centrifuge, similar to What's In My Pot (WIMP), as described with the long reads. The reads identified as *E. coli* by taxon number (see script) were extracted and saved in paired fastq files.

*De novo* assembly of short reads alone was performed using the CLC Genomics Workbench (v20.0.2; Qiagen, Germantown, MD). Several *de novo* hybrid assemblies using both total and *E. coli* extracted (binned) long and short reads (from the same sample and dilution for both sequencing technologies) were generated using default parameters for SPAdes v3.13.1 (Antipov et al., 2016), Unicycler v0.4.8 (Wick et al., 2017), and OPERA_MS v19.07.01 (Bertrand et al., 2019). The assembled contigs were classified by taxonomy by Kraken2 (Wood et al., 2019) using GalaxyTrakr (Gangiredla et al., 2021). The presence of the complete genome and synteny was checked using the Mauve genome aligner (Darling et al., 2004) and compared to the reference genome generated previously using nanopore long reads (Maguire et al., 2021a).

### Closure of high-quality EDL933_2 genome by long- and short-read sequencing

For bioinformatic quality control purposes, we generated a high-quality closed genome of the strain (EDL933_2) used in the artificial contamination studies. The long-read sequencing as well as the assembly using only long reads were reported earlier (Maguire et al., 2021a). The short reads were generated as above using a MiSeq Illumina sequencer. The high-quality EDL933_2 genome was generated by a hybrid assembly using long and short reads with Unicycler v0.4.8 (Wick et al., 2017), as described previously (Maguire et al., 2021b).

### *In silico* serotyping

Batch screening of the *de novo* assemblies was performed to analyze the major serotype present in each sample using Ridom SeqSphere+ software v7.0.6 (Ridom, Münster, Germany) using the genes deposited in the Center for Genomic Epidemiology (http://www.genomicepidemiology.org/services/) for *E*. *coli* as part of their web-based tool, SerotypeFinder 2.0 (https://cge.food.dtu.dk/services/SerotypeFinder/).

### *In silico* identification of virulence genes

The *de novo* assemblies were batch screened for virulence genes using Ridom SeqSphere+ using the genes deposited in the NCBI Pathogen Detection Reference Gene Catalog (https://www.ncbi.nlm.nih.gov/pathogens/isolates#/refgene/) and described in the study of Gonzalez-Escalona and Kase (2019).

### Phylogenetic relationship of the strains by cgMLST analysis

The phylogenetic relationship of the strains was assessed by a core genome multilocus sequence typing (cgMLST) analysis using Ridom SeqSphere+. The genome of O157:H7 strain Sakai (NC_002695) was used as a reference. A cgMLST phylogenetic analysis of 4,200 O157:H7 genomes available at NCBI using 4,651 loci from Sakai showed that 17 strains clustered highly together to the strain EDL933_2 used in this study (this includes the four sample duplicates/strains and the EDL933_2 hybrid assemblies from this study). We used Nei's DNA distance method (Nei et al., 1983) for calculating the matrix of genetic distance, taking into consideration only the number of same/different alleles in the core genes. A neighbor-joining (NJ) tree using pairwise ignoring missing values and the appropriate genetic distances was built after the cgMLST analysis. cgMLST uses the allele number of each locus for determining the genetic distance and builds the phylogenetic tree. The use of allele numbers reduces the influence of recombination in the dataset studied and allows for fast clustering determination of genomes.

### Metagenomic data and high-quality EDL933_2 genome accession numbers

The Illumina metagenomic sequence data from this study are available in GenBank under BioProject number PRJNA639799. The genome for EDL933_2 was deposited under accession number CP120944-CP120946.

## Results

### Microbial community identification by MiSeq sequencing

To be able to perform phylogenic and SNP analysis on the sequenced *E. coli* O157 strain, short-read sequencing by Illumina MiSeq technology was performed with the same DNA extractions used previously (Maguire et al., 2021a). Artificial contamination of STEC-negative agricultural water enrichment with 10-fold dilutions of the *E. coli* O157:H7 EDL933_2 variant produced final concentrations of 7 × 10^8^ CFU/ml (Water + Ecoli1) to 7 × 10^4^ CFU/ml (Water + Ecoli5) plus a negative control sample of only enriched agricultural water (Water). As mentioned in the Materials and Methods section, each sample was run in duplicate in two different MiSeq sequencing runs, and the output resulted in an average of 8.7 million reads and 2.25 Gb yield per sample (Table 1). The sequenced reads for each sample were classified by taxonomy using Centrifuge (Kim et al., 2016), which is the basis for the EPI2ME WIMP workflow used for the taxon classification of the nanopore reads (Maguire et al., 2021a). We report the taxa accounting for >1% abundance of the total unique reads.

**Table 1:** MiSeq sequencing and assembly summary statistics.

**Sample name**	**EDL933_2 concentration (CFU/ml)**	**Yield (Gb)**	**Total reads**	**Contigs**
Water A^a^	None	2.5	11,004,158	14,355
Water B^a^	None	1.6	6,933,448	12,974
Water + Ecoli1A^a^	7.00E+08	1.5	6,240,592	11,455
Water + Ecoli1B^a^	7.00E+08	1.8	8,243,832	10,015
Water + Ecoli2A^a^	7.00E+07	1.9	8,357,928	13,509
Water + Ecoli2B^a^	7.00E+07	2.1	9,562,322	11,235
Water + Ecoli3A^b^	7.00E+06	2.0	8,513,148	15,133
Water + Ecoli3B^b^	7.00E+06	2.1	8,772,570	15,138
Water + E coli4A^b^	7.00E+05	2.3	9,936,638	13,851
Water + E coli4B^b^	7.00E+05	2.2	9,561,540	13,431
Water + Ecoli5A^b^	7.00E+04	2.1	8,983,248	13,448
Water + Ecoli5B^b^	7.00E+04	2.1	9,125,196	13,486

Replicates for each sample are listed as A or B.

a Samples in run 1.

b Samples in run 2.

**Figure 1:**
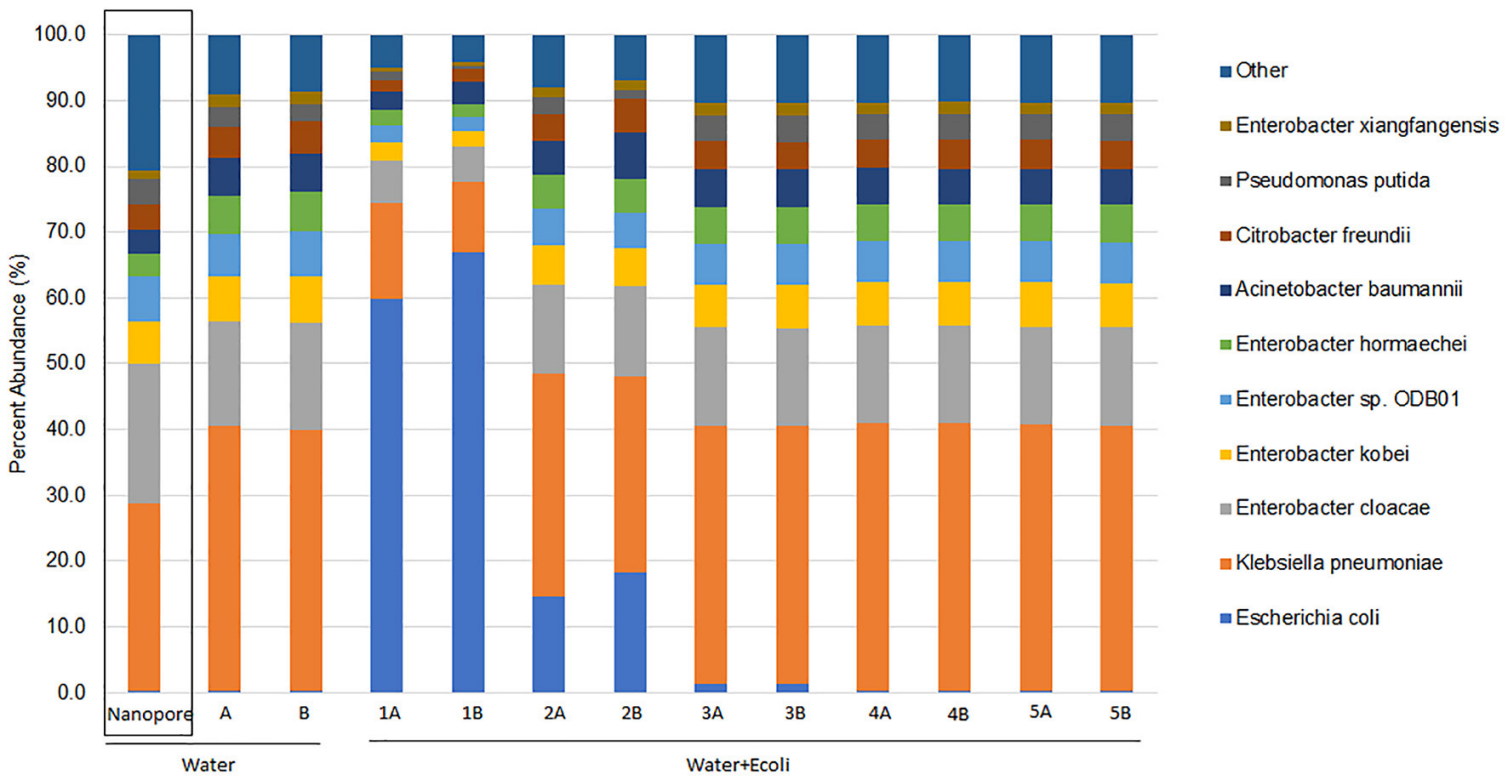
Relative abundance of bacterial species associated with pre-harvest agricultural water with and without artificial *Escherichia coli* EDL933_2 contamination from MiSeq sequencing read output. Enriched pre-harvest agricultural water (Water) was artificially contaminated with 10-fold dilutions of *E. coli* EDL933_2 (+Ecoli) with a starting concentration of 7 × 10^8^ CFU/ml (Water + Ecoli1). Extracted DNA was previously sequenced by nanopore, and bacterial species were identified using the EPI2ME WIMP workflow (Water Nanopore) (Maguire et al., 2021a). MiSeq sequencing was performed in duplicate, and read output was classified by taxonomy using centrifuge (Kim et al., 2016). Bacterial species contributing more than 1% of the unique identified reads are shown, and the sum of the remaining species identified is included as “Other.”

Centrifuge analysis of MiSeq short reads identified a highly diverse bacterial community. Most of the sample was comprised of the same nine highly abundant species as previously determined by nanopore and subsequent WIMP analysis but with slightly different proportions (Figure 1). The duplicate MiSeq samples demonstrated approximately the same percent abundance. The nine bacterial species and their approximate proportions identified by MiSeq sequencing and Centrifuge analysis in the background water sample were *Klebsiella pneumoniae* (40%), *Enterobacter cloacae* (16%), *Enterobacter kobei* (7%), *Enterobacter* sp. ODB01 (6.5%), *Enterobacter hormaechei* (6%), *Acinetobacter baumannii* (5.8%), *Citrobacter freundii* (4.9%), *Pseudomonas putida* (3%), and *Enterobacter xiangfangensis* (1.9%). The percentage of species abundance (% of total reads) classified by Centrifuge was very similar between nanopore and MiSeq data for the water sample, except for *K. pneumoniae* and *E. cloacae* (Figure 1).

### Limit of detection and assembly using short-read MiSeq sequencing alone

To better understand the depth of sequencing and microbial community characterization of enriched agricultural water metagenomic samples, we aimed to establish the limits of detection and assembly of the MiSeq technology alone and in combination with the previous nanopore data. The agricultural water sample determined by the FDA BAM Chapter 4A method to be STEC-negative was confirmed as such by the Centrifuge taxonomy classification analysis of the short-read MiSeq output for duplicates of the water sample (WaterA and WaterB). There were 12,672 (0.17%) and 8,362 (0.18%) reads, respectively, belonging to generic *E. coli* in this sample, and none of these reads matched STEC O157:H7.

However, for the spiked samples, the percentage abundance of *E. coli* reads was proportional to the added STEC concentration, ranging from ~60% in Water + Ecoli1 (A: 2,628,675 reads and B: 3,724,064 reads) to 0.2% in Water + Ecoli5 (A: 10,827 reads and B: 10,985 reads). While Centrifuge does not perform strain-level identification, we determined the limit of detection for *E. coli* O157 using MiSeq reads as samples containing the “*Escherichia* Stx1 converting phage.” In samples Water + Ecoli4A and B, 34 and 19 total reads were identified as *Escherichia* Stx1 converting phage, respectively. Therefore, the limit of detection of *E. coli* O157 in MiSeq reads was determined to be 10^5^ CFU/ml when multiplexed. If only a single sample is run, then not only the detection limit but also the cost per sample will be higher.

To assess the limit of assembly, we closed the EDL933_2 genome obtained from the hybrid assembly of nanopore long reads and Illumina MiSeq short reads. We used this genome as a reference to ensure the accuracy of our *in silico* analyses to detect serotype (O157:H7) and virulence gene profiles, including *stx1a* and *eae* gamma-1 (Table 2). The genome assembly limit is the minimum number of reads necessary to produce either a complete, closed metagenome-assembled genome (MAG) with 20× coverage or a complete, fragmented genome that ensures accurate serotyping and a complete virulence profile. We performed a *de novo* assembly with the CLC Genomics Workbench on the total MiSeq read output, resulting in an average of 13,170 contigs per sample (Table 1). As expected, only the O9 serotype was detected in WaterA and WaterB samples, and no *stx, eae*, or additional virulence genes were found. The O157:H7 serotype, *stx1a, eae* gamma-1 genes, and complete virulotype were detected in samples Water + Ecoli1A and B and Water + Ecoli2A and B. At lower spiked levels, Water + Ecoli3A and B, only the un-spiked O9 serotype could be detected, and the *stx* and *eae* gene identification was lost. It was possible to detect some virulence genes but with inconsistent sample accuracy (Table 2). Therefore, the limit of assembly of MiSeq sequencing using all reads was determined to be 10^7^ CFU/ml.

**Table 2:** Serotype and virulence gene identification in *de novo* assembled contigs from Illumina MiSeq sequencing reads.

**Sample^a^**	**ST**	**Serotype^b^**	**Stx type**	**Eae**	***ehxA***	***espA***	***espB***	***espF***	***espJ***	***espK***	***espP***	***tccP***	***etpD***	***nleA***	***nleB***	***nleC***	***tir***	***katP***	***pssA***	***toxB***
EDL933_2^c^	11	O157:H7	1a	Gamma-1	+	+	+	+	+	+	+	+	+	+	+	+	+	+	+	+
Water A	–	O9	–	–	–	–	–	–	–	–	–	–	–	–	–	–	–	–	–	–
Water B	–	O9	–	–	–	–	–	–	–	–	–	–	–	–	–	–	–	–	–	–
Water + Ecoli1A	11	O157:H7	1a	Gamma-1	+	+	+	–	+	+	+	–	+	+	+	+	+	+	+	+
Water + Ecoli1B	11	O157:H7	1a	Gamma-1	+	+	+	–	+	+	+	–	+	+	+	+	+	+	+	+
Water + Ecoli2A^d^	11	O157:H7	1a	Gamma-1	+	+	+	–	+	+	+	–	+	+	+	+	+	+	+	+
Water + Ecoli2B^d^	unk	O157:H7	1a	Gamma-1	+	+	+	–	+	+	+	–	+	+	+	+	+	+	+	+
Water + Ecoli3A	unk	O9	1a	–	+	–	+	–	–	–	–	–	+	–	–	–	–	+	–	–
Water + Ecoli3B	unk	O9	–	–	–	+	+	–	+	–	–	–	+	–	+	+	+	+	–	–

a CFU/ml levels of EDL933_2 inoculation can be found in Table 1.

b *In silico* serotype using genes defined by the Center for Genomic Epidemiology at the technical University of Denmark (DTU) (https://cge.cbs.dtu.dk/services/SerotypeFinder/).

c *Escherichia coli* O157:H7 EDL933_2 variant strain reference hybrid assembly for serotyping and virulotyping.

d Complete, fragmented genome assembly limit.

unk—incomplete profile.

### Precision metagenomic assembly limit using *E. coli*-binned MiSeq reads

We previously demonstrated that we were able to improve the limit of fragmented MAG assembly and generate a completely closed MAG by binning the nanopore reads belonging to a particular species, which we termed precision metagenomics (Maguire et al., 2021a). This process used a custom Python script to extract the WIMP-identified “*E. coli*” reads into a separate fastq file used for *de novo* assembly. Therefore, we developed a similar custom script using the Centrifuge taxonomic classifier to extract the *Escherichia* MiSeq paired-end reads into two fastq files (R1 and R2; Supplementary Note). *Escherichia* identified reads decreased according to the spiked concentration, with 3,666,510 and 5,335,057 *E. coli* reads in Water + Ecoli1A and B samples, while samples Water + Ecoli4A/B and 5A/B contained similar read numbers than the negative control WaterA and B samples (Table 3). *De novo* assembly with the CLC Genomics Workbench produced between 319 and 2,884 contigs from the binned reads. Water + Ecoli1A and B and Water + Ecoli2A and B contigs produced assemblies with at least 48× coverage and 5.6 Mb, which is the size of the EDL933_2 genome (5.6 Mb). However, at lower spiked concentrations (Water + Ecoli3 and below), the coverage was below 10× and the complete genome could not be assembled (Table 3). Therefore, the limit of assembly using precision metagenomics was determined to be 10^7^ CFU/ml, the same as using the total read output.

**Table 3:** *De novo* assembly statistics from *Escherichia coli*-binned Illumina MiSeq sequencing reads.

**Sample name^a^**	***Escherichia coli* reads**	**Contigs**	**Coverage (X)^b^**	**Total size (Mb)**
Water A	45,312	388	2	0.48
Water B	69,430	319	3	0.41
Water + Ecoli1A	3,666,510	358	157	5.51
Water + Ecoli1B	5,335,057	418	216	5.45
Water + Ecoli2A^c^	1,201,634	450	48	5.60
Water + Ecoli2B^c^	1,658,476	555	65	5.60
Water + Ecoli3A	137,880	2,852	6	4.35
Water + Ecoli3B	140,346	2,884	6	4.37
Water + Ecoli4A	65,145	662	3	0.74
Water + Ecoli4B	61,901	613	3	0.71
Water + Ecoli5A	56,493	353	3	0.49
Water + Ecoli5B	57,789	377	3	0.50

a CFU/ml levels of EDL933_2 inoculation can be found in Table 1.

b Coverage is relative to the EDL933_2 variant strain genome size of ~5.6 Mb.

c Complete, fragmented genome assembly limit.

### Limit of assembly using the hybrid assembly of binned nanopore reads and binned MiSeq reads

We have previously demonstrated improved assembly output by using a hybrid assembly technique with a combination of nanopore long reads and MiSeq short reads (Gonzalez-Escalona and Sharma, 2020). Numerous software programs have been developed for this purpose, and each can produce slightly different assemblies. Therefore, we tested three different hybrid assemblers: Unicycler (Wick et al., 2017), SPAdes (Antipov et al., 2016), and OPERA-MS (Bertrand et al., 2019). Due to the large metagenomic input of the MiSeq reads, the hybrid assemblers with *E. coli*-binned nanopore reads and the total MiSeq output performed poorly (data not shown). Consequently, we used the *E. coli*-binned extracted MiSeq reads and binned nanopore reads to generate hybrid assemblies using the same three hybrid assemblers (Tables 4, 5). *In silico* analysis of WaterA and B samples from each of the three assemblers detected only the O9 serotype (Table 4), and no virulence genes were identified (data not shown). For the spiked samples, the O157:H7 serotype, *stx1a* gene, and *eae* gamma-1 gene could be detected with a concentration as low as 10^6^ CFU/ml assemblies generated with Unicycler and OPERA-MS. While the O157:H7 serotype could be identified at 10^5^ CFU/ml with the SPAdes assembly, the *stx* and *eae* gene identification was less reliable between samples (Table 5).

**Table 4:** Spades hybrid assembly statistics using *Escherichia coli*-binned nanopore and MiSeq sequencing reads.

**Sample^a^**	**ST**	**Serotype**	**Stx type**	**Eae type**	**Contig no. (O157 chromosome and plasmid)**	**Percent EDL933_2 genome assembled**
WaterA	–	O9	–	–	571	0%
WaterB	–	O9	–	–	472	0%
Water + Ecoli1A	11	O157:H7	1a	Gamma-1	484 (13 and 1)	100%
Water + Ecoli1B	11	O157:H7	1a	Gamma-1	256 (37 and 1)	100%
Water + Ecoli2A	11	O157:H7	1a	Gamma-1	313 (19 and 1)	100%
Water + Ecoli2B	11	O157:H7	1a	Gamma-1	353 (35 and 1)	100%
Water + Ecoli3A	–	O157:H7	1a	Gamma-1	559 (197 and 2)	95%
Water + Ecoli3B	–	O157:H7	1a	Gamma-1	572 (204 and 6)	95%
Water + Ecoli4A	–	O9	–	–	939	0%
Water + Ecoli4B	–	O9	–	–	938	0%

^a^CFU/ml levels of EDL933_2 inoculation can be found in Table 1.

**Table 5:** Virulence gene identification in *de novo*, hybrid assembled contigs from binned nanopore reads and binned Illumina MiSeq sequencing reads.

	**EDL933_2**	**Unicycler**								**Spades**								**OPERA-MS**							
		**Water** + **Ecoli**								**Water** + **Ecoli**								**Water** + **Ecoli**							
		**1A**	**1B**	**2A**	**2B**	**3A**	**3B**	**4A**	**4B**	**1A**	**1B**	**2A**	**2B**	**3A**	**3B**	**4A**	**4B**	**1A**	**1B**	**2A**	**2B**	**3A**	**3B**	**4A**	**4B**
*astA*	+	+	+	+	+	+	+	–	–	+	+	+	+	+	+	–	–	+	+	+	+	+	+	–	–
*ehxA*	+	+	+	+	+	+	+	–	–	+	+	+	+	+	+	+	+	+	+	+	+	+	+	+	+
*espA*	+	+	+	+	+	+	+	–	–	+	+	+	+	+	+	+	+	+	+	+	+	+	+	–	–
*espB*	+	+	+	+	+	+	+	–	–	+	+	+	+	+	+	+	+	+	+	+	+	+	+	–	–
*espF*	+	+	+	+	+	+	+	–	–	+	+	+	+	–	–	+	–	+	+	+	+	–	+	–	–
*espJ*	+	+	+	+	+	–	+	–	–	+	+	+	+	+	+	–	–	+	+	+	+	+	+	–	–
*espP*	+	+	+	+	+	+	+	–	–	+	+	+	+	+	+	–	+	+	–	–	+	+	+	+	+
*tccP*	+	+	–	+	+	–	+	–	–	+	+	+	+	+	+	–	–	+	+	+	+	+	+	–	–
*etpD*	+	+	+	+	+	+	+	–	–	+	+	+	+	+	+	+	+	+	+	+	+	+	+	+	+
*gad*	+	+	+	+	+	+	+	–	–	+	+	+	+	+	+	+	+	+	+	+	+	+	+	–	+
*iha*	+	+	+	+	+	+	+	–	–	+	+	+	+	+	+	+	+	+	–	+	+	+	+	–	–
*iss*	+	+	+	+	+	+	+	–	–	+	+	+	+	+	–	–	–	+	+	+	+	+	+	–	–
*nleA*	+	+	+	+	+	–	+	–	–	+	+	+	+	+	+	+	–	+	+	+	+	+	+	–	–
*nleB*	+	+	+	+	+	+	+	–	–	+	+	+	+	+	+	–	–	+	+	+	+	+	+	–	–
*nleC*	+	+	+	+	+	+	+	–	–	+	+	+	+	+	+	–	–	+	+	+	+	+	+	–	–
*tir*	+	+	+	+	+	+	+	–	–	+	+	+	+	+	+	+	+	+	+	+	+	+	+	+	+
*katP*	+	+	+	+	+	+	+	–	–	+	+	+	+	+	+	–	–	–	–	+	+	+	+	+	+
*toxB*	+	+	+	+	+	+	+	–	–	+	+	+	+	+	+	+	+	+	+	+	+	+	+	+	+
*ecf1*	+	+	+	+	+	+	+	–	–	+	+	+	+	+	+	–	+	+	–	+	+	+	+	+	+
IEE	+	+	+	+	+	+	+	–	–	+	+	+	+	+	+	–	+	–	+	+	+	–	+	–	+
*espK*	+	+	+	+	+	+	+	–	–	+	+	+	+	+	+	–	+	+	+	+	+	+	+	–	–
*pssA*	+	+	+	+	+	+	+	–	–	+	+	+	+	+	+	–	+	+	+	+	+	+	+	–	–
*air*	+	+	+	+	+	+	+	–	–	+	+	+	+	+	+	+	+	+	+	+	+	+	+	–	–

CFU/ml levels of EDL933_2 inoculation can be found in Table 1.

Virulence gene identification, however, demonstrated marked differences in the three assemblies produced. *In silico* analysis of the Unicycler assembly was able to detect all of the virulence genes at a concentration of at least 10^7^ CFU/ml, with the exception of Water + Ecoli1B, in which *tccp* was not found. Similarly, analysis of the SPAdes assembly indicated that a complete, fragmented MAG could be generated with a concentration of at least 10^7^ CFU/ml. OPERA-MS, however, generated assemblies from which all virulence genes could not be identified consistently at any concentration (Table 5). Therefore, a limit of complete, fragmented assembly using a hybrid assembly of *E. coli*-binned nanopore reads and *E. coli*-binned MiSeq read output was established as 10^7^ CFU/ml with the SPAdes assembler. A complete, closed MAG could not be generated at any concentration using any of the assemblers tested here.

### Phylogenetic analysis of the SPAdes hybrid assemblies by cgMLST analysis

**Figure 2:**
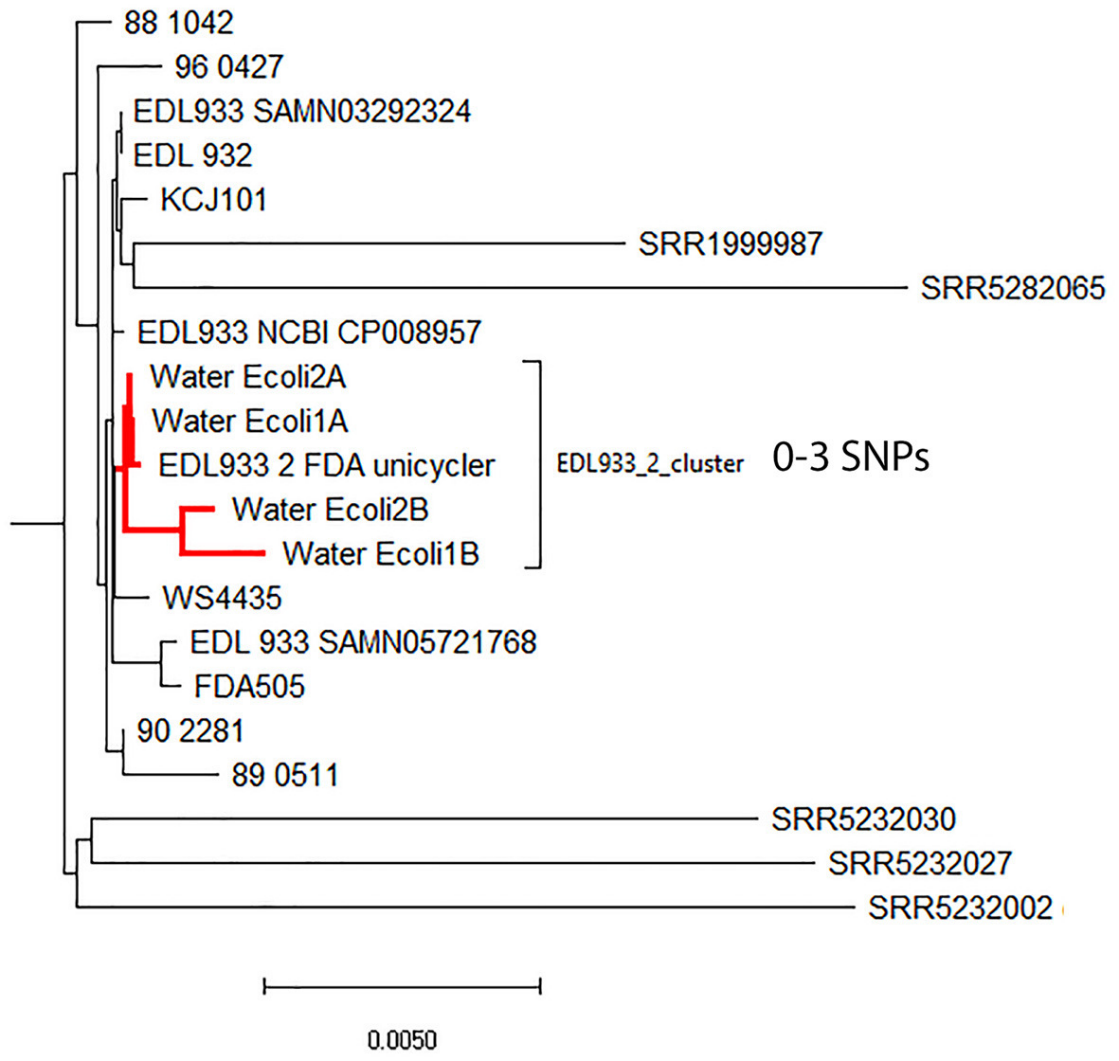
Neighbor-joining phylogenetic tree generated by a cgMLST analysis of the hybrid assemblies (HAs) for the different spiking levels and 4,200 other O157 *Escherichia coli* strains (available at NCBI). The genome of *E. coli* O157:H7 strain Sakai (NC_002695) was used as a reference. The final cgMLST analysis was based on 4,651 shared loci among those strains. The HA for the different spiking levels in this study is in red.

The phylogenetic relationship among the different hybrid assemblies obtained for each spiking level, that of strain EDL933_2, and other O157:H7 isolates was assessed by a cgMLST analysis using the genome of *E. coli* strain Sakai (NC_002695). The initial analysis failed because there were other *E. coli* contigs that interfered with the analysis (data not shown). To correct and eliminate these potential contigs present in the final assembly, we used the genome assembled using only the binned nanopore reads as a reference to re-organize the contigs from the hybrid assemblies using the Mauve aligner (Darling et al., 2004). By using this method, we were able to correctly identify the contigs belonging to EDL 933_2 in the spiked hybrid assembly. The new phylogenetic analysis using the filtered EDL933_2 contigs showed that the hybrid assemblies for spiked levels above 10^7^ CFU/ml were placed correctly in the phylogenetic tree (Figure 2). Hybrid assemblies at 10^6^ CFU/ml or below were too fragmented, and the phylogenetic analysis lost precision, with the selected contigs falling outside the EDL933_2_FDA cluster.

## Discussion

Most food safety laboratories are relying heavily on different methods of recovery and identification of foodborne pathogens to facilitate the fast identification and source tracking of specific strains during foodborne outbreaks. Among those foodborne outbreaks of high importance are those caused by STECs in produce. Due to the importance of agricultural water to produce and food safety, accurate detection and classification of STECs potentially present is of major importance, especially during a foodborne outbreak. Current methods for strain identification (usually by SNP or MLST analysis) during a foodborne outbreak require sequencing of pure strains by either short (usually Illumina) or long reads (usually PacBio or Oxford Nanopore Technologies). However, to obtain a single pure colony strain, there are several steps, such as initial detection [usually by real-time PCR (qPCR)] and extensive selective plating, before a single colony can be obtained. This is a time-consuming process that only provides confirmation of an isolate after almost 2 weeks of labor, when the associated contaminated product might be out of the market chain. By combining qPCR and short-read and long-read metagenomic analysis of the enrichment, we can definitively detect an STEC isolate and characterize its virulence potential in 3–4 days. While this will not replace eventual confirmation by microbiological methods, this reduces the time for a prospective corrective measure by a complete week.

A previous study has determined the limits of detection and assembly for STECs in enriched water using long reads (nanopore) (Maguire et al., 2021a). However, the same limits for Illumina MiSeq have not been determined yet. Our first goal from this study was to determine those. The importance of these determinations is paramount to evaluating the feasibility of the use of any technique to support outbreak investigations. Short reads have been used for culture-independent surveillance and have shown promising results in retrieving STEC, *Salmonella*, and *L. monocytogenes* genomes in as short as 24 h (Leonard et al., 2015, 2016; Ottesen et al., 2020; Saltykova et al., 2020; Townsend et al., 2020; Buytaers et al., 2021; Commichaux et al., 2021; Zhang et al., 2021; Vorimore et al., 2023). However, those genomes were in high concentration in the sample (~80%−90%), indicating that, when levels are ~10^8^ CFU/ml or higher, short reads will perform correctly (the target strain will be easily identified if only one strain is present). The main pitfall of the use of those short reads will be that the genome assembly will still be composed of many non-contiguous contigs, and many important traits or markers of the genome might be lost.

In this study, we started by taxonomically classifying the microorganisms present in the enriched water sample used as a negative control for the presence of STEC O157:H7 using short reads (Illumina MiSeq). In general, the proportions were similar to what was obtained previously for the same samples using long reads (nanopore) (Maguire et al., 2021a), except for two microorganisms (*K. pneumoniae* and *E. cloacae*). This was unexpected, and the explanations could be as follows: (1) Illumina reads are shorter, which might lead to misclassification and overrepresentation of certain taxa; (2) while both analyses use Centrifuge, the RefSeq databases used might include some differences in classification and could contribute to the different classifications; (3) MiSeq loads only part of the library; (4) the efficiency in library preparations can vary with the GC content of the microorganisms; and (5) since most taxonomy classifiers rely on read numbers matching to the organism, in the case of short reads, some bigger bacterial genomes (>5 Mb) might appear to be more represented in the sample than smaller genomes. There is a need for further experiments to test the accuracy of strain-level identification by Centrifuge in metagenomics samples between nanopore and MiSeq sequencing outputs.

We continued by determining empirically the detection and assembly limits of the STEC strain EDL933_2 using Illumina MiSeq reads. The main difference with the experiment in our previous study, using long reads (nanopore), was that we multiplexed six samples per MiSeq run instead of a single sample per run. This allows us to perform sequencing of the same sample per duplicate per run. The fact that we used six samples per MiSeq run was because the limit of detection or assembly from metagenomic samples using that equipment was currently unknown. MiSeq detection limit using all reads was determined to be 10^5^ CFU/ml for STECs (when using six samples in a V3 cartridge), and the assembly limit was ~10^7^ CFU/ml to obtain a fragmented MAG. Even after binning the reads matching to *E. coli*, the assembly limit remained the same, although with a lower number of contigs (Tables 1, 3). When using a single sample per run, the estimated detection and assembly limits should therefore be six times higher than what we observed. The cost for a nanopore run is approximately USD$ 1,100, while an Illumina MiSeq run without multiplexing is approximately USD$ 1,500. All these variables (price, limits of detection, and assembly) should be taken into consideration when planning metagenomic studies using a MiSeq platform, specifically if multiplexing samples are used in a single run.

Assembling STEC genomes is a very complicated matter, and if the STECs are in mixed culture, it is even more difficult (Buytaers et al., 2021; Maguire et al., 2021a; Jaudou et al., 2022). We have previously shown that a completely closed STEC O157:H7 MAG can be recovered from enriched samples by sequencing using long reads (nanopore) at 10^7^ CFU/ml and higher levels (Maguire et al., 2021a). However, this completely closed MAG was not of high quality to be used for high-precision SNP analysis that could reveal the correct placement of the genome in a phylogenetic tree (results not shown). Therefore, we intended to test whether, by using a hybrid assembly approach, we could obtain similar results (completely closed MAG from a metagenomic sample) and with enough quality to be used for SNP analysis for source tracking as commonly used in outbreak investigations (Hoffmann et al., 2016; Crowe et al., 2017; Brown et al., 2019; Saltykova et al., 2020; Haendiges et al., 2021; Vorimore et al., 2023). For the hybrid assembly, we selected three known software (SPAdes) and pipelines (Unicycler and OPERA-MS). As mentioned in the Results section, neither of the assemblers performed adequately to meet our needs (recovery of the completely closed or fragmented O157:H7 STEC MAG) when using the complete datasets using default parameters. That is not to say that they performed inadequately for MAG hybrid assemblies overall for the entire metagenomic sample. That was not part of our tests. Considering that, the binned *E. coli* reads extracted from both long and short-read datasets were used instead for testing the performance of the hybrid assemblers. They performed with varying degrees of effectivity with SPAdes producing the better outcome (Table 5). They all showed the same limit of assembly (10^7^ CFU/ml) but failed to recover a completely closed MAG for any of the spiking levels. This was contrary to what was observed when using long reads alone (Maguire et al., 2021a), where a completely closed O157:H7 STEC MAG was recovered for spiking levels above 10^7^ CFU/ml. A similar observation when using hybrid assemblies for metagenomic sequences was observed for *L. monocytogenes* in enrichments from ice cream (Commichaux et al., 2021), where the hybrid assemblers using short reads as the first step of the assembly resulted in more accurate assemblies but were more fragmented and failed to recover a completely closed genome.

Another important consideration is that OPERA-MS requires 4 Gb of output data for samples with low complexity and 10 Gb for samples with high complexity. In our case, we had ~2 Gb per sample, which might explain why our OPERA-MS assemblies were the worst performers of the three tested ones, suggesting that a single sample should be run in a MiSeq system if a better hybrid assembly is necessary to obtain better or more complete MAG assemblies. However, a different system with higher output per sample could be used (i.e., NextSeq 2000 or another short-read sequencer) to fulfill the software pipeline requirements, albeit with higher expenses per run. If a simple estimation of taxonomic diversity is desired, the MiSeq approach might suffice if lower depth per sample is not a problem.

One of the reasons why we picked OPERA-MS for the hybrid assembly was that the authors claimed that their pipeline was able to detect strains of the same organism in the metagenomic sample (Bertrand et al., 2019). This is a very important and attractive feature that is desired when performing metagenomic analysis of enriched food or water samples. When food samples or water samples are analyzed, single colonies are picked and then tested for specific targets and sequenced (Walters et al., 2013). This process might miss some other co-occurring strains present in the sample, and those strains might be of lower prevalence (Commichaux et al., 2021). However, Unicycler was not designed for the assembly of metagenomic samples (Wick et al., 2017), but since we extracted the *E. coli* reads from both long- and short-read sequencing data for each individual sample, we expected it to perform successfully when used for a single organism.

Most of the current use of WGS in bacterial-associated diseases is focused on the sequencing of isolated microorganisms from selective culture plates (Hoffmann et al., 2016; Gobin et al., 2018). Most clinical and diagnostic laboratories are moving toward the use of culture-independent diagnostic tests (CIDTs) to quickly identify within minutes the etiological agent causing the illness. However, contrary to the culture method, which takes longer and requires more effort, a physical isolate is not produced at the end of the diagnosis. The lack of a physical isolate dramatically affects how public health agencies can properly identify potential outbreak clusters in outbreak investigations. This, in turn, has a negative impact on public health and increases the time to resolve ongoing or potential outbreaks (Carleton et al., 2019). A comparable situation occurs when trying to isolate foodborne bacteria from foods for surveillance or outbreak investigations, such as STECs, which take ~2 weeks to obtain a single isolate before doing WGS on that isolate and complete the outbreak investigation (Maguire et al., 2021a). Consequently, there is a renewed effort to move to culture-independent subtyping and outbreak investigations for pathogen infections in both clinical and food investigations (Carleton et al., 2019; Peña-Gonzalez et al., 2019; Maguire et al., 2021a). These methods or approaches need to be rapid, cheap (95–300 USD), and highly accurate (Carleton et al., 2019). In a previous publication, we were able to obtain completely closed STEC genomes from enriched samples (using a metagenomic approach); however, the technology employed (ONT) produced genomes that were not suitable for source tracking using an SNP approach. In this study, by adding Illumina reads and assembling the data using both Illumina and ONT data using a hybrid approach, we were able to obtain fragmented STEC O157:H7 MAGs with enough quality and accuracy to be able to be placed in the correct cluster when analyzed against 4,200 O157:H7 genomes available at NCBI (Figure 2). Both replicates for each spike level (1e^8^ and 7e^7^ CFU/ml) clustered together with the genome generated for the completely circular closed genome (EDL933_2 FDA Unicycler) spiked strain and differed among them by a maximum of three SNPs.

Current developments in ONT sequencing are showing promising results, with newer sequencing kits producing data of higher quality (Q20+) (preliminary unpublished data by authors). However, more studies and advances are still needed to make it suitable to be used as an alternative method to culture-based approaches, as well as the creation of improved assemblers for that kind of data.

## Conclusion

Overall, we tested the limits of detection and assembly for EDL933 O157:H7 in enriched irrigation water using a shotgun short-read sequencing approach (MiSeq Illumina). In our previous study, the nanopore sequencing detection and assembly limits were determined to be 10^3^ and 10^5^ CFU/ml, respectively. In this study, we showed that, for MiSeq sequencing, the detection and assembly limits when using six samples per MiSeq V3 kit were two 10-fold lower at 10^5^ and 10^7^ CFU/ml, respectively, when compared to nanopore sequencing. Furthermore, short-read sequencing (MiSeq Illumina) of the highest level of EDL933_2 spiking of 7 × 10^8^ CFU/ml did not result in a completely closed genome as observed with the nanopore sequencing in our previous study. Nevertheless, the completely fragmented genome or fragmented MAG obtained with levels above 10^7^ CFU/ml with short reads was enough to make a complete characterization of the STEC strain, including serotype and virulotype. In this study, we ran six samples per single MiSeq reagent cartridge, and the analysis of the results provided vital information regarding how many samples could be run successfully in a single cartridge and their coverage depth, answering several unknowns about the limit of detection and assemblies for samples with varying degrees of abundance in the sample. For example, if your target of interest in your sample is low, then we suggest running a single sample per run or sequencing them in other instruments (i.e., NextSeq 2000) that can produce higher output data. Still, the cost of your sampling/analysis ratio must be taken into consideration as well. Contrary to what we were expecting, a hybrid assembly approach did not produce a completely closed genome even at the highest concentration (7 × 10^8^ CFU/ml), highlighting the complexity of STEC genome assemblies. Nonetheless, these fragmented MAG hybrid genomes clustered with the correct samples in an SNP tree of more than 4200 STEC O157:H7 genomes. Further studies using nanopore sequencing kits that show promising results in read quality and accuracy could help in solving these issues of closing high-quality STEC genomes from metagenomic samples to be used for outbreak investigations while the single purified strain is isolated for regulatory reasons. The impact of this single *in silico* purified strain would be remarkable, allowing to speed up the process of pulling potentially contaminated products from the shell earlier, reducing the number of illnesses associated with that product, and helping in root case analysis of the possible sources of contamination of such products. We must stress though that additional advances in the technology (more samples and output per run) and price reduction per run will make this approach more accessible for use in routine food testing at most laboratories.

## Data availability statement

The datasets presented in this study can be found in online repositories. The names of the repository/repositories and accession number(s) can be found at: https://www.ncbi.nlm.nih.gov/genbank/, CP120944-CP120946 and PRJNA639799.

## Author contributions

NG-E and MMag conceived and designed the experiments and wrote the manuscript. NG-E, MMag, and MMam analyzed the data. NG-E, MMag, MMam, MA, EB, and SM contributed to reagents, materials, and analysis tools. MMag, MA, ST, EB, and NG-E revised and drafted the study.

## Footnotes

^1^https://www.fda.gov/media/102633/download and https://www.fda.gov/media/83177/download.

^2^https://www.fda.gov/food/outbreaks-foodborne-illness/environmental-assessment-factors-potentially-contributing-contamination-romaine-lettuce-implicated and https://www.fda.gov/food/outbreaks-foodborne-illness/investigation-summary-factors-potentially-contributing-contamination-romaine-lettuce-implicated-fall.
