# Supplementary material for: Precision metagenomics sequencing for food safety: hybrid assembly of Shiga toxin-producing Escherichia coli in enriched agricultural water
Source: Front Microbiol. 2023 Aug 31;14:1221668. doi: 10.3389/fmicb.2023.1221668 (PMC10500926; doi:10.3389/fmicb.2023.1221668)
Supplement: Supplementary file 1 [file Data_Sheet_1.docx]

Supplementary Material

Precision metagenomics sequencing for food safety: hybrid assembly of Shiga toxin-producing *Escherichia coli* in enriched agricultural water

**Meghan Maguire1, Padmini Ramachandran1, Sandra Tallent^1^, Mark Mammel2, Eric W. Brown^1^, Marc Allard^1^, Steven M. Musser^1^, Narjol González-Escalona^1^,***

*** Correspondence:** Corresponding author. narjol.gonzalez-escalona@fda.hhs.gov. Mailing address, Center for Food Safety and Applied Nutrition, Food and Drug Administration, 5001 Campus Drive, College Park, MD 20740, USA.

**Supplementary Note.** Custom python script to extract reads for a desired taxon from Centrifuge MiSeq classified data.

import gzip

#---------------------------------------------------------

#replace the string in parenthesis with your own centifuge tabular file

centrifuge_file = 'Centrifuge_on_1E1B_MiSeq__Output.tabular'

#-------------------------------------------

#specify your preferred filter ids here

ids = set([561,562,564,10710,10677,10742,29252,37554,40631,194948,208962,245685,337828,343516,399183,669875,906668,906669,1124654,1124655,1131317,1141141,1150869,1216926,1229753,1327956,1446490,1458706,1527519,1541883,1649239,1649240,1660365,1837867,1720495,1391428,10679,66711,10678,355246,1054834,1237364,1430444])

#-------------------------------------

#replace the string after the equal sign with your own fastq file

data_files = ["1E1-B_S6_L001_R1_001.fastq.gz", "1E1-B_S6_L001_R2_001.fastq.gz"]

#------------------------------------------

out_files = ["out1E1BMiSeq_R1.fastq", "out1E1BMiSeq_R2.fastq"]

fastq_lines = 4

keep_set = set()

print("reading centrifuge file")

with open(centrifuge_file, 'r') as f:

for line1 in f:

row = line1.split()

if len(row) > 2:

readid = row[0]

try:

taxid = int(row[2])

if taxid in ids:

keep_set.add(readid)

except ValueError:

taxid = 0

# don't handle the exception

f.close()

nreads = len(keep_set)

print(nreads, "reads selected")

print("saving fastq files")

for i in range(len(data_files)):

file_in = data_files[i]

file_out = out_files[i]

count = 0

outputDataFile = open(file_out, 'w')

keep = False

mod4 = 0 #assume four line format for fastq files

#with open(file_in, 'r') as f:

with gzip.open(file_in, 'rt') as f:

for line1 in f:

if mod4 == 0:

# read id in fastq file

# @M03101:6:000000000-CVNB2:1:1101:18905:1000 1:N:0:1

# read id in centrifuge file

# M03101:6:000000000-CVNB2:1:1101:21670:1015

pos = line1.find(' ')

if pos > 0 and line1[1:pos] in keep_set:

keep = True

count += 1

else:

keep = False

if keep:

outputDataFile.write(line1)

mod4 = (mod4 + 1) % fastq_lines

f.close()

outputDataFile.close()

print(count, "reads saved")
